# Editorial: Plasticity of GABAergic synapses

**DOI:** 10.3389/fncel.2015.00262

**Published:** 2015-07-07

**Authors:** Andrea Barberis, Alberto Bacci

**Affiliations:** ^1^Neuroscience and Brain Technologies, Post-synaptic Mechanisms of GABAergic Transmission, Fondazione Istituto Italiano di TecnologiaGenova, Italy; ^2^Inserm U 1127, Centre National de la Recherche Scientifique UMR 7225, Sorbonne Universités UPMC Paris 06, UMR S 11Paris, France; ^3^Institut du Cerveau et de la Moelle ÉpinièreParis, France

**Keywords:** inhibitory plasticity, ILTP, inhibitory synapse, GABAA receptors, inhibitory microcircuits

For long time, plasticity of brain circuits has been hypothesized to mainly rely on the flexibility of glutamatergic excitatory synapses, whereas inhibitory synapses have been assumed to be relatively invariant. Based on this view, inhibition should be exclusively modulated by the differential glutamatergic-driven activation of a highly diverse population of inhibitory interneurons displaying specific temporal dynamics and selective innervation patterns. However, it has been demonstrated that inhibitory synapses undergo several forms of plasticity, thus providing an additional source of versatility to the regulation of the neuronal network and the emergence of complex brain states.

The cellular and molecular mechanisms occurring at inhibitory synapses during the induction/expression of inhibitory short- and long-term synaptic plasticity are now beginning to be unraveled. At the presynaptic side, retrograde synaptic messengers modulate GABA release (Mendez and Bacci, 2011; Iremonger et al., 2013; Lourenco et al., 2014; Younts and Castillo, 2014), whereas postsynaptic plasticity typically involves changes in the number/gating properties of post-synaptic GABAA receptors (Kurotani et al., 2008; Houston et al., 2009; Luscher et al., 2011; Petrini et al., 2014; Flores et al., 2015). In addition, acute or chronic alterations of intracellular chloride concentration modulate the driving force of GABAergic currents and the subunit composition of GABAA receptors (Woodin et al., 2003; Raimondo et al., 2012; Succol et al., 2012).

The 14 articles presented in this ebook (including hypothesis and theory, minireviews, reviews, and original research articles) cover the mechanisms of inhibitory synaptic plasticity, at the molecular and microcircuit levels. Zacchi et al. (2014) focus on the signaling pathways controlling the phosphorylation state of gephyrin, a key scaffold protein at inhibitory synapses responsible for the synaptic clustering of both glycine and GABAA receptors. By considering the synapse as a highly dynamic element, Petrini and Barberis (2014), review the recent literature addressing the role of protein diffusion in the reorganization of the inhibitory postsynaptic density during inhibitory synaptic plasticity. A similar conceptual approach, based on the analysis of receptor dynamics, has been adopted by Muir and Kittler (2014) to investigate inhibitory plasticity in relation to GABAA receptor diffusion at inhibitory synapses located in the axon initial segment. This original research article reports that chronic depolarization increases the lateral mobility of GABAA receptors and reduces the size of post-synaptic GABAA receptor clusters, thus critically interfering with neuronal excitability. Hirano and Kawaguchi (2014) review another form of postsynaptic inhibitory plasticity observed at cerebellar synapses formed by stellate cells onto Purkinje cells. This inhibitory long-term potentiation involves the CaMKII-dependent increase of GABAA receptor signaling through direct GABAA receptor phosphorylation and/or promoted surface delivery via a GABARAP-dependent mechanism. In their original article, Gao et al. (2014) further address the molecular mechanisms of the aforementioned long-term inhibitory plasticity at cerebellar Purkinje cells. They report that the pathway of iLTP induction critically depends on the coordinated action of both αCaMKII and βCaMKII isoforms, and is modulated by the activation of GABAB receptors. Flores et al. (2015) provide a broad yet detailed analysis of the molecular organization of inhibitory post-synaptic density. In addition, they highlight the formation and elimination of GABAergic synapses as an important source of inhibitory synaptic plasticity. The mini review by Maguire (2014) examines the plasticity of inhibition in response to acute and chronic stress involving region-specific changes of GABAA receptor subunit expression and alterations of the chloride gradient. Moreover, Dr. Maguire reports that stress acts as a metaplastic switch by enabling iLTP at parvocellular neuroendocrine cells (PNCs). Mapelli et al. (2015) provide a comprehensive overview of diverse forms of plasticity at specific cerebellar sub-circuits, introducing the concept of the coordination between excitatory and inhibitory plasticity for correct circuit functioning. In their minireview, Chevaleyre and Piskorowski (2014) highlight the importance of short- and long-term changes of inhibitory synaptic strength in tuning the threshold for the induction of excitatory plasticity. In addition, they discuss how plasticity of glutamatergic synapses onto PV+ interneurons shapes inhibition at hippocampal microcircuits. Pallotto and Deprez (2014) analyze the influence of inhibition in adult neurogenesis in the olfactory bulb and dentate gyrus, by discussing the role of GABAergic signaling in the development and plasticity of adult-born neurons. In their comprehensive review Griffen and Maffei (2014) examine different forms of pre- and post-synaptic inhibitory plasticity occurring at diverse somatosensory cortex interneuron subtypes and discuss the role of such plasticity in sensory cortical activity.

Synaptic signaling does not only depend on pre- or post-synaptic determinants but is also shaped by the dynamics of neurotransmitter in the synaptic cleft. The minireview and the hypothesis and theory by Scimemi (2014a,b) propose the intriguing idea that changes of GABA transporters activity may modulate GABAergic responses. In particular, by exploiting a computer modeling approach, Dr. Scimemi validates the hypothesis that the density, distribution and lateral mobility of GABA transporters affect the GABA concentration sensed by postsynaptic GABAA receptors.

In addition to synaptic inhibition, tonic inhibition produced by the persistent activation of extrasynaptic GABAA receptors is crucial for the tuning of neuronal excitability. Recent evidence demonstrates that also tonic inhibition is plastic. The original article by Barth et al. (2014) illustrates that the ovarian cycle is associated with variations of expression of GABAA receptors containing the “tonic” δ-subunit, both in hippocampal principal cells and interneurons. Interestingly, such plasticity modulates γ-oscillations, thus representing a possible determinant for altered memory and cognitive performance observed during ovarian cycle.

The ability of inhibitory synapses to undergo plasticity emphasized in this ebook raises important questions. First, what are the specific molecular mechanisms of inhibitory plasticity at synapses formed by different interneuron subtypes? Second: how is plasticity orchestrated at both excitatory and inhibitory synapses? In keeping with this, how do different forms of excitatory and inhibitory plasticity co-exist? Do variations of both excitation and inhibition strength occur in parallel/homestatic (Froemke et al., 2007; Xue et al., 2014; Flores et al., 2015), independent (Lourenco et al., 2014), or opposite fashions (Petrini et al., 2014). Are these different “plasticity modes” dependent on the stimulus pattern, specific spatial distributions of synapses and/or time points after plasticity induction? What are the behavioral and cognitive correlates of these different forms of plasticity?

Answering these questions will contribute in redefining the excitation to inhibition balance (E/I) as a “dynamic” activity-dependent determinant for the functioning of brain microcircuits.

## Conflict of interest statement

The authors declare that the research was conducted in the absence of any commercial or financial relationships that could be construed as a potential conflict of interest.
